# Inferring genome-scale rearrangement phylogeny and ancestral gene order: a *Drosophila *case study

**DOI:** 10.1186/gb-2007-8-11-r236

**Published:** 2007-11-08

**Authors:** Arjun Bhutkar, William M Gelbart, Temple F Smith

**Affiliations:** 1 BioMolecular Engineering Research Center, Boston University, Cummington St, Boston, MA 02215, USA; 2 Department of Molecular and Cellular Biology, Harvard University, Cambridge, MA 021383, USA

## Abstract

A simple, fast, and biologically inspired computational approach for inferring genome-scale rearrangement phylogeny and ancestral gene order has been developed. This has been applied to eight *Drosophila *genomes. Existing techniques are either limited to a few hundred markers or a small number of taxa. This analysis uses over 14,000 genomic loci and employs discrete elements consisting of pairs of homologous genetic elements. The results provide insight into evolutionary chromosomal dynamics and synteny analysis, and inform speciation studies.

## Abstract 2

A simple, fast, and biologically-inspired computational approach to infer genome-scale rearrangement phylogeny and ancestral gene order has been developed and applied to eight Drosophila genomes, providing insights into evolutionary chromosomal dynamics.

## Background

Chromosomal rearrangements have been studied in *Drosophila *since the early 20th century, originally via optical observation of banding patterns [1-4]. Chromosomal inversions have been inferred from such observations as well as from other genomic marker pairs [2,5-7]. These inversions and clusters of banding patterns have also been used to study evolutionary history [8,9], adaptation, and speciation [10,11]. More recently, the identification and analysis of gene synteny (conserved blocks of ordered genes) has been used to infer evolutionary rearrangements and relationships among organisms from bacteria [12] to *Drosophila *[13] and mammals [14]. The primary motivation for this work is to provide a fast computational method to derive phylogenetic relationships, and to estimate rearrangement counts and ancestral gene order for large datasets, while overcoming the limitations of current gene order based methods described below. These methods either do not converge on a solution for large datasets or are limited by execution speed and input data size to a few hundred markers or a small number of taxa.

There have been a number of modern approaches to full-genome comparative analysis and gene order analysis [14-18]. Parsimonious methods based on gene order analysis usually begin with a search for homologous genes and the identification of syntenic gene clusters. They have generally been limited by the need to compensate, insofar as possible, for homolog uncertainty in the presence of paralogs, and for missing data in assembly gaps. Such approaches usually build a graphical representation to map the synteny linkage between pairs of chromosomes. These graphical representations can be processed computationally via various algorithmic approaches [19-23] to find the minimum number and specific types of genetic events that would result in the observed mapping, thus providing an estimate of the distance between genomes. Methods focusing on gene order and content data have been investigated in detail [23,24] with a focus on the computational issues involved therein. The general computational problem of reconstructing a phylogeny from gene order data is NP-hard [25-27] and various heuristics have been employed [23].

Studying genome rearrangements is an important tool that aids in the understanding of evolutionary events. Previous approaches using pairs of chromosome bands [9], multidirectional chromosome painting [28] and pairs of adjacent genes to study rates of genome shuffling [29] have shown how rearrangements affect genome organization during evolution. This provides some of the motivation for the method presented here.

Comparative analysis of insect genomes is expected to yield significant insights into evolution, development, and regulation [30]. With the availability of a large number of fully sequenced genomes, particularly from closely related species, there is now a need to revisit such methodologies with the aim of reconstructing detailed genome-wide evolutionary histories. The recently sequenced genomes of a large number of fruit fly (*Drosophila*) species (Drosophila 12 Genomes Consortium, 2007) and other insects provide an ideal data set for this purpose. The currently assumed phylogenetic relationships between various fly species [31,32] involve species thought to have diverged from 5 million to about 50 million years ago. Research on *D*. *melanogaster *(*Dmel*) has provided a wealth of tools and resources [33] over the years, including the well annotated *D*. *melanogaster *genome sequence [34].

Chromosomal translocations are rare in *Drosophila *species [35]. Most genes are restricted to the same arm or Muller element [36] with reshuffling along the arm due to paracentric inversions. This potentially simplifies the analysis of rearrangements. While gene translocation via retrotransposition [37,38] does occur (Bhutkar A, Russo S, Smith TF, Gelbart WM, Genome Scale Analysis of Positionally Relocated Genes, *Genome Research (in press)*), it appears to be rare [34]. Over the course of the 20th century, *Drosophila *phylogeny was estimated using a number of high-level methods, such as morphological analysis, geographical distribution, limited genetic analysis, and from sequence variation of a small set of genes. The techniques and results presented in this study support the recently updated phylogenetic grouping of *Drosophila yakuba *(*Dyak*) and *Drosophila erecta *(*Dere*), provide a validation of the assumed *Drosophila *phylogeny for the remaining species, and estimate the number of fixed chromosomal rearrangement breaks based on genome-scale analysis involving over 14,000 (over 32,000 including outgroup species) precise molecular markers. While accommodating gene translocation between arms, and paracentric and pericentric inversions, this approach uses neighboring gene pairs (NGPs) across multiple closely related species to infer evolutionary relationships, a rearrangement phylogeny, and ancestral syntenic arrangements. The fundamental biologically inspired idea is that inversions are rare events, pairs of adjacent genetic loci observed in multiple species probably existed in their common ancestor, and each inversion disrupts two pairs of neighboring genetic elements and creates two new pairs. Essentially, the likelihood of two independent inversions in disjoint lineages creating the same pair of adjacent genetic loci is low. This approach is a significant advance over existing techniques in its speed, its ability to handle large datasets that were previously unmanageable, and in its ability to process preliminary genome assembly data - as outlined in the Discussion section. The results place *Drosophila *inter-species rearrangement relationships on a solid footing. Furthermore, chromosomal inversions have been mapped to specific branches of the tree for all species, and previously unknown *Drosophila *ancestral gene arrangements have been inferred. This also quantifies and highlights particular lineages and species that have undergone a high level of chromosomal rearrangements, thus supplying critical information for speciation studies.

## Results

**Figure 1:**
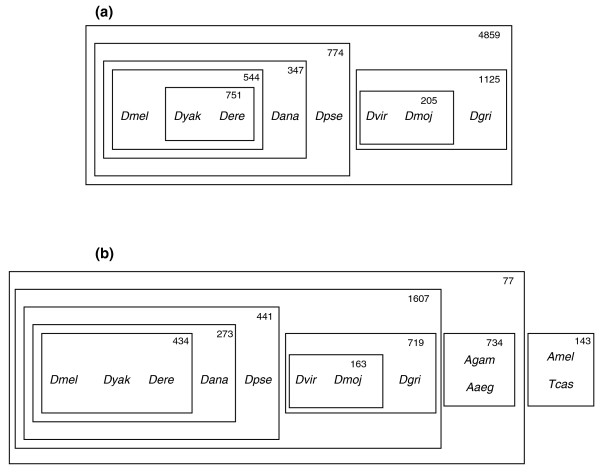
Partitioning of various *Drosophila *species and outgroup species (*Anopheles gambiae *(*Agam*), *Aedes aegypti *(*Aaeg*), *Apis mellifera *(*Amel*), and *Tribolium castaneum *(*Tcas*)) based on 'exclusively shared NGPs' (NGPs found in each species in a clustered group and not found in any species outside this group - see Materials and methods). A box around a pair of species, a cluster and a species, or two clusters, signifies that they are inferred to be grouped together in the phylogeny. Numbers denote the actual number of 'exclusively shared NGPs' unique to each cluster. **(a) **Arm-indexed clustering within genus *Drosophila*. Genes with orthologs in all genus *Drosophila *species (see Materials and methods for species' names) are chosen to form NGPs. This clustering reveals subgenus *Drosophila*, subgenus *Sophophora *and *melanogaster *subgroup species to be distinct clusters. This binary partitioning validates the placement of *Dyak *(see text) and agrees with the currently understood phylogenetic relationships between other *Drosophila *species (see Discussion for details). **(b) **Relaxed clustering without arm indexing for NGPs, in order to include outgroup species that differ in chromosomal architecture (see Materials and methods). The set of common genes between all species, including outgroup species, is used to derive NGPs. Relaxing arm indexing results in loss of signal within the closely related *melanogaster *subgroup species (*Dmel*, *Dyak*, *Dere*) where *Dmel *+ *Dere*, *Dyak *+ *Dere*, and *Dmel *+ *Dyak *are weak clusters with 16, 15, and 9 exclusively shared NGPs, respectively. See Discussion and Materials and methods for details.

Utilizing 8,967 high-confidence genes common to all *Drosophila *species (Additional data file 1) resulted in 14,947 arm-indexed NGPs (Additional data file 2) across all *Drosophila *species, excluding outgroup species. Clustering these arm-indexed NGPs to maximize 'exclusively shared NGPs' (see Materials and methods) resulted in species partitioning for initial phylogenetic analysis (Figure 1a and Additional data file 4). See Materials and methods for details on this similarity maximization metric and the motivation behind it. These phylogenetic relationships validate the currently accepted placement of *D. yakuba *on the evolutionary tree [39-46], which is also supported by a shared meta-centric inversion with *D. erecta *[47].

**Figure 2:**
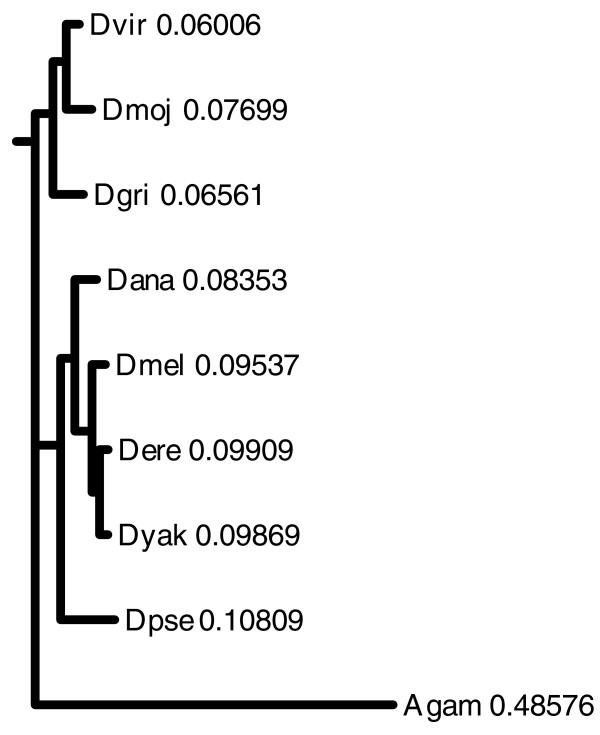
Maximum likelihood gene tree generated with PHYLIP version 3.65 using amino acid sequences for proteins SRP54 and SRP19 from various genus *Drosophila *species and *Anopheles gambiae *(Agam) as the outgroup species. Data for the tree is also provided in Additional data file 9. The tree has been artificially rooted with outgroup species (Agam). Numbers reflect the relative arm lengths from this root. Species within subgenus *Drosophila *(*Dvir*, *Dmoj*, *Dgri*) show lower overall average branch length than species within subgenus *Sophophora*, similar to Figure 3.

To test this method with distant outgroup species, a set of high-confidence common genes across *Drosophila *species and four outgroup species was chosen while relaxing the arm-indexing requirement for NGPs in order to allow for varying chromosomal architecture of outgroup species. This resulted in a set of 4,085 genes and 19,416 NGPs, which were clustered using the same similarity maximization metric (Figure 1b and Additional data file 5). A loss of signal for closely related species (*Dmel*, *Dyak*, *Dere*) is noticeable due to the lack of arm-indexing. See Discussion for details. For validation, a maximum likelihood gene tree was generated using a set of universal eukaryotic genes (*SRP54 *and *SRP19*) thought to be under minimal species-specific selection. The resulting gene tree (Figure 2) has an identical topology to the partitioning (Figure 1a).

To infer *Drosophila *ancestral adjacencies, the set of common genes across *Drosophila *species was chosen (8,967 genes), the arm indexing criterion was relaxed to allow for varying chromosome architecture, and four outgroup species were added to form the set of NGPs. This resulted in a total of 32,154 NGPs (Additional data file 3) out of which 14,162 NGPs were contributed by one or more *Drosophila *species. The count of *Drosophila *NGPs is down from 14,947 arm-indexed NGPs to 14,162 as a result of relaxing the arm-indexing requirement.

**Figure 3:**
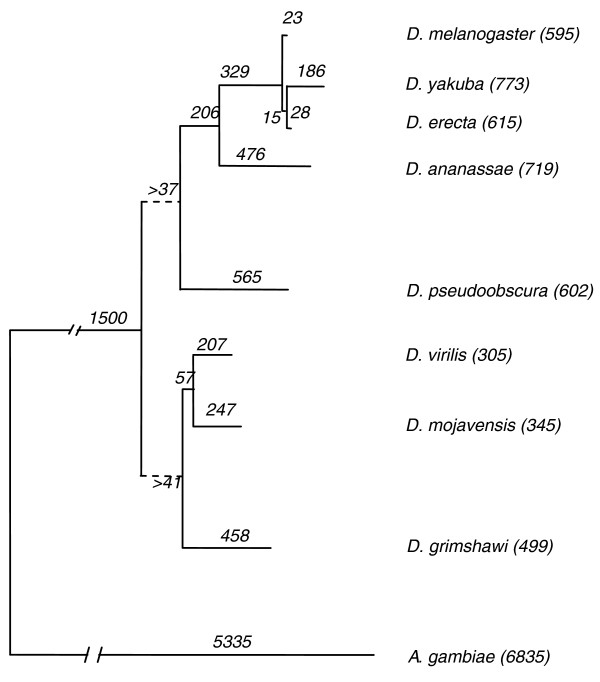
Rearrangement phylogeny for genus *Drosophila*. The number along each branch of the tree shows the probable number of fixed rearrangement breaks inferred along that evolutionary branch. Each inferred rearrangement break corresponds to the disruption of a gene pair (NGP) that was inferred to exist in the immediate ancestor. Consequently, it includes macro and micro syntenic disruptions. See Materials and methods for details on the handling of ambiguous cases. Rearrangement breaks are assumed to occur as a result of chromosomal inversion events. Estimates for inversion counts can be computed from these data as outlined in the Materials and methods. The total number of inferred fixed rearrangement breaks for each genus *Drosophila *species, from the *Drosophila *root, is mentioned alongside the species name. *Anopheles gambiae *(shown), *Aedes aegypti*, *Apis mellifera*, and *Tribolium castaneum *are also used as outgroup species. Subgenus *Drosophila *species show lower overall average branch lengths than subgenus *Sophophora *species. Dashed lines at the subgenus *Sophophora *and subgenus *Drosophila *nodes reflect the loss of genus-specific NGP signal at the genus *Drosophila *root, which is only partially compensated for by distant outgroup species. See Discussion for details.

Starting with the NGP phylogeny inferred earlier, and performing an iterative walk down and up this implied phylogeny (Figure 1a), estimates for the number of fixed rearrangement breaks along each branch of the tree are calculated (Figure 3) as outlined in the Materials and methods section. For a given node, the rearrangement phylogeny estimates a lower bound for the number of disruptions of NGPs that existed at the immediate ancestor. Ambiguous cases are handled as discussed in Materials and methods with evidence from outgroup species, wherever applicable. An estimate of the inversion count can be computed from a rearrangement phylogeny as the number of inversion events that resulted in the observed rearrangements (each inversion disrupts two ancestral gene pairs and creates two new pairs).

Comparison with known rearrangements in the *eve *region of *Drosophila *[42] shows that the adjacency between genes CG2328 and CG2331 is captured in three species (*Dmel*, *Dere*, *Dyak*) and is absent in the other species, as expected. CG2328 is adjacent to CG30421 in the other species and this adjacency is inferred to be ancestral as evidence for it straddles the *Drosophila *root, pointing to a rearrangement in the branch leading to *Dmel*, *Dere*, and *Dyak*. Further, a comparison with analysis of rearrangements reported earlier in the *lab-pb *region [43] shows that the *lab-pb *neighborhood is captured correctly as an adjacency in *Dmel *and *Dpse*. It is also inferred to be an ancestral adjacency with evidence from subgenus *Sophophora *species, which is in line with earlier analysis [43].

**Figure 4:**
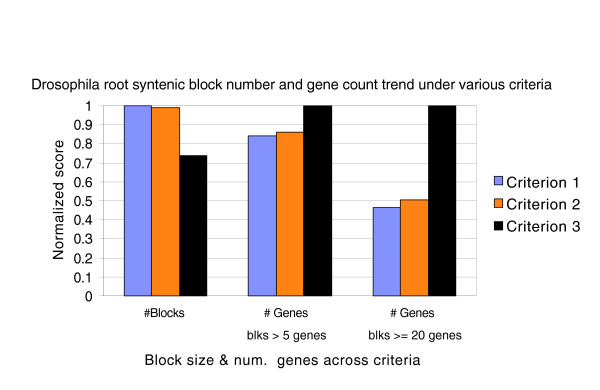
Comparison between number of syntenic blocks and total number of genes in syntenic blocks of various sizes at the *Drosophila *root. Values are normalized between 0 and 1 with the maximum value set to 1. The x-axis shows various criteria based on the different relaxed assumptions discussed in the text. Criterion 1: first-pass syntenic blocks. Criterion 2: results of further merging based on outgroup evidence. Criterion 3: further merging of syntenic blocks based on relaxed assumption of bridging blocks using genes on block edges paired in at least one fly species. As additional evidence is incorporated using relaxed assumptions, blocks are merged into longer chains, which results in a lowering of the total number of syntenic blocks (1: 1,029 blocks, 2: 1,018 blocks, 3: 758 blocks). Correspondingly, the number of genes in larger blocks increases (for blocks >5 genes in size: 1: 5,532 genes, 2: 5,656 genes, 3: 6,576 genes; for blocks ≥20 genes in size: 1: 1,230 genes, 2: 1,329 genes, 2,638 genes).

A comparison of the relative number of ancestral syntenic blocks and gene count in syntenic blocks under various assumptions used in this method is shown (Figure 4). The distribution of ancestral syntenic block sizes, in terms of gene count, at the root of the genus *Drosophila *tree computed by this method under various criteria is presented (Table 1, Additional data files 6 and 7). The largest ancestral syntenic block at the genus *Drosophila *root has 61 genes under the most relaxed assumptions (criterion 3). Of the 13,706 euchromatic genes annotated in FlyBase release 4.3 [44], filtering out genes based on lack of strong homologous placements in one or more species and other criteria (embedded genes, assembly gaps, and so on), a set of 8,967 common genes (Additional data file 1) was used in this analysis. This is a conservative set that can be expanded as better homology data become available across species. A little over 73% (62% for criterion 1; 63% for criterion 2) of these 8,967 *D. melanogaster *annotated genes were placed in ancestral syntenic blocks of size greater than five genes, and approximately 30% (14% for criterion 1; 15% for criterion 2) were placed in blocks of size 20 genes or more at the root of the genus *Drosophila *tree under the most relaxed assumptions (criterion 3). In the context of rearrangement activity within *Drosophila *species, of the 8,967 common genes, 3,691 (41%) genes were seen only in two NGPs and the rest were observed in three or more NGPs across all species.

**Table 1:** Distribution of syntenic block sizes (≥3 genes) at the root of the *Drosophila *tree under various relaxed criteria

	No. of blocks		
	
Syntenic block size (no. of genes)	Criterion 1	Criterion 2	Criterion 3
3	283	279	162
4	119	113	57
5	137	136	67
6	86	82	52
7	73	71	55
8	54	59	53
9	34	30	29
10	39	35	33
11	36	33	27
12	26	28	19
13	22	23	28
14	22	24	27
15	16	15	18
16	11	13	14
17	9	12	10
18	9	10	9
19	6	5	7
20	7	7	9
21	6	5	7
22	5	5	7
23	1	3	6
24	4	4	6
25	2	2	4
26	4	4	5
27	2	2	6
28	2	2	2
29	4	3	5
30	1	2	4
31	0	1	5
32	3	2	5
33	2	3	2
35	0	0	2
36	1	1	2
38	0	0	1
39	1	1	1
40	1	1	2
41	0	1	1
43	0	0	1
44	0	0	1
46	0	0	1
47	0	0	2
48	1	1	1
51	0	0	1
54	0	0	1
61	0	0	1

Note that criterion 2 is weaker than criterion 1 and criterion 3 includes the weakest assumptions. Criterion 1: first-pass syntenic blocks. Criterion 2: result of bridging syntenic blocks with genes on block edges paired using outgroup species evidence. Criterion 3: further merging of syntenic blocks based on relaxed assumption of bridging blocks using genes on block edges paired in at least one fly species. See Additional data files 6 and 7 for gene composition of blocks.

## Discussion

In contrast to existing approaches, this method provides a computationally fast technique that infers phylogenetic relationships between a given set of species and calculates rearrangement counts and probable ancestral syntenic blocks. The genus *Drosophila *phylogenetic relationships derived using arm-indexed NGPs (Figure 1a) match previously assumed relationships [31,32], and lend support to the clustering of *D. yakuba *with *D. erecta *as opposed to being clustered with *D. melanogaster*. This had been a source of debate in the *Drosophila *community [37-46], with small-scale evidence supporting the alternative hypothesis until it was resolved recently [39]. This clustering is also supported by the fact that both *D. yakuba *and *D. erecta *share a pericentric inversion between Muller elements B and C, indicating a shared evolutionary event distinct from *D. melanogaster *[47]. Relaxing the arm-indexing criteria to include outgroup species (Figure 1b) expands the set of NGPs (over 32,000) but results in loss of signal between closely related species that share chromosomal architecture and might differ only slightly in their gene order through transposition events. Arm-indexing proves to be a valuable tool in the phylogenetic analysis of closely related species that might share most of their paracentric inversions (due to a common lineage) and differ only slightly in gene order as a result of a small number of arm transpositions or pericentric inversions.

The total rearrangement counts from the root of the *Drosophila *tree to each fly species indicate that subgenus *Drosophila *(*D. virilis *(*Dvir*), *D. mojavensis *(*Dmoj*), *D. grimshawi *(*Dgri*)) species show lower overall average branch lengths than subgenus *Sophophora *species, which is similar to the relative branch lengths in the *SRP *gene tree (Figure 2). The rearrangement count for *Anopheles gambiae *would be higher if the distribution of shared genes across different arms is taken into account as separate events. Additional analysis of rearrangement rates [45] using the results of the NGP method is the subject of further study. In order to account for differing qualities of species assemblies, this method identifies all genes on assembly scaffold edges and on singleton scaffolds. As a result, breaks in gene pairs at assembly scaffold edges do not result in over-counting rearrangement events due to low level of assembly quality. Probable assembly errors can be identified via adjacent blocks that violate arm indexing with lack of supporting evidence from other species, barring species-specific cases. Furthermore, an indication of assembly gaps in a given species can be derived from the number of genes missing in that species, but present in two or more neighboring species, assuming a low number of single taxon gene loss events in closely related species.

The distribution of syntenic block sizes at the root of the *Drosophila *tree (Figure 4, Table 1) illustrates the incorporation of sequentially relaxed assumptions in the computation of syntenic blocks. The first-pass syntenic blocks (criterion 1) are bridged and extended using outgroup evidence and subsequently using bridging pairs that occur in at least one species anywhere in the *Drosophila *tree. Each relaxation leads to joins of progressively lower confidence. In the case of criterion 3 (Table 1), there may exist conflicts between two possible joins. However, these relaxed criteria are in line with our earlier assumption about the low probability of identical NGPs being created independently in different species. The number of syntenic blocks starts out with 1,029 blocks in the initial analysis and then decreases (down to 1,018 blocks with outgroup evidence and to 758 blocks with evidence from any one *Drosophila *species) as blocks are merged into longer blocks by incorporating additional evidence (Figure 4). The total gene count across variously sized syntenic blocks also increases with the addition of further evidence. The distribution of block sizes (Table 1) shows how the chaining of syntenic blocks results in larger blocks with an increased gene count as the assumptions are relaxed.

The identification of genes involved in multiple dissimilar NGPs at a rate above a threshold would give a probable set of genetic loci in the neighborhood of rearrangement hotspots. An analysis of the association between these probable hotspots and transposable elements in various species can be undertaken as such elements are characterized across different *Drosophila *species. The distribution of transposable elements on *Drosophila *chromosomes is known to be non-random [48,49]. Transposable elements, repeats and breakpoint motifs have been implicated in generating chromosomal inversions in *Drosophila *by some studies [13,50-53]. Some studies indicate that rearrangement junctions might not be significantly enhanced for transposable elements [13] and that these elements might be over-represented in chromosomal areas with lower recombination rates [48,49].

Although the simple computational approach presented here uses homologous protein coding genes and corresponding NGPs, the method is applicable to a wide range of homologous genome markers. This method falls under the broad class of parsimonious gene-order approaches [23] with a few differences. It relies on the fundamental biologically inspired idea that inversions are rare events, pairs of adjacent genetic loci observed in multiple species probably existed in their common ancestor, and each inversion disrupts two pairs of neighboring genetic elements and creates two new pairs. The use of a higher order construct like arm-indexed NGPs for phylogenetic clustering and a two stage tree traversal procedure to infer ancestral gene synteny are other key features. The first stage of this approach, inferring phylogenetic relationships through maximizing gene pair similarity (as opposed to the traditional distance measure used by other techniques), is motivated by the assumption that if species share a NGP, it is the result of an inversion event along a shared lineage that resulted in the creation of that NGP that has not been disrupted by additional events (that is, ancestral gene pair conserved in extant species). Additionally, the likelihood of finding the same NGP in other species that do not share that lineage is rare. The clustering of certain species to the exclusion of others is based on the maximization of 'exclusively shared NGPs' (NGPs found in all species in a cluster and not found in any species outside this cluster - see Materials and methods). This allows for the method to extract a strong signal to cluster species into smaller groups although they might share other ancestral NGPs in common with species that are evolutionarily farther away. This is particularly evident in the arm-indexing of NGPs to form sub-clusters within a group of closely related species. The limits of this approach would be reached if single taxon inversion events dominate (and lineage-specific inversion events are rare), resulting in homoplasy in the inversion dataset. For a given set of species, if the level of inversion homoplasy in the dataset rivals the number of 'exclusively shared NGPs' that cluster sub-groups of species together, loss of the NGP signal would render this method ineffective. The second stage of this approach, inferring rearrangement counts, is motivated by the fact that ancestral NGPs can be inferred using the principle that NGPs seen in species across both sides of a node existed at that node with high probability and that NGP disruptions are the result of shared (given rarity of inversions) or single taxon inversion events that disrupt NGPs. The same principles are also used in the inference of ancestral syntenic blocks where evidence to chain syntenic blocks comes from the derived ancestral NGPs and outgroup conservation of NGPs assuming that those pairs existed at the common ancestor rather than being derived independently a result of identical inversions across multiple lineages.

Using these simple strategies, this method has the advantage of simplicity, speed, missing data tolerance and the flexibility to exploit various levels of biological assumptions. In order to overcome some of the speed and data size limitations of existing approaches, we make a number of practical assumptions and use decision-making strategies as discussed in the Materials and methods section. The implementation avoids the need for more complex heuristics for NP-hard problems that are often employed [19-23,25], at least for relatively closely related species. It appears quite insensitive to assembly incompleteness and probable errors.

Compared to simple parsimony approaches that rely on sequence divergence (nucleotide or amino acid), gene order based approaches explore a much larger search space. We contrasted this approach with three existing parsimonious gene order techniques: BPAnalysis [54], GRAPPA [25], and MGR [55]. BPAnalysis attempts to solve the NP-hard breakpoint median problem using the traveling salesman problem (TSP) heuristic to minimize the breakpoint distance between gene orders. Solving the TSP for all nodes across all possible trees is exponential in the number of genomes and number of genes. BPAnalysis works for gene orders on uni-chromosomal genomes and trees of eight or fewer leaves [23]. GRAPPA is an optimized re-implementation of the BPAnalysis 'breakpoint distance' metric with algorithmic improvements for execution speed, data size, and inclusion of inversion distance. It utilizes the TSP heuristic for breakpoint medians and a branch-and-bound strategy for inversion medians. GRAPPA speeds up the BPAnalysis implementation significantly and can solve the breakpoint phylogeny or the inversion phylogeny problem; however, it remains an exponential time algorithm for breakpoint phylogeny. It is limited to a few hundred genes per genome and works for uni-chromosomal genomes. Other approaches based on GRAPPA include GRIMM [56], which works on pairs of genomes. MGR, which uses GRIMM for distance computation, uses a 'reversal-distance' minimization strategy and is applicable to multi-chromosomal genomes. It proposes the identification of 'good reversals' that reduce the reversal distance between sets of three genomes and their ancestor for median inference. MGR is better in its speed and ability to handle multiple genomes when compared with GRAPPA; however, it has been tested only on a few hundred markers across genomes [55]. In contrast to these techniques, the approach presented here handles multi-chromosomal datasets with thousands of markers.

We used the most widely used existing implementation of parsimonious gene order based analysis, GRAPPA, to do a run-time comparison. GRAPPA has exponential runtime in the number of genomes and the number of genes. Even after limiting the input dataset to one *Drosophila *chromosome arm (about 1,650 common genes per species, as opposed to over 8,000 common genes and over 14,000 NGPs across the genome in our analysis and over 32,000 NGPs including outgroup species), GRAPPA did not complete and did not suggest a candidate phylogeny despite running over six hours. Our clustering approach derives NGPs and suggests a candidate phylogeny within a few minutes and our heuristic derives ancestral syntenic blocks in approximately 10 minutes for a significantly larger dataset on the same dedicated Pentium 4 laptop computer.

To further test our approach, we used a test dataset of mitochondrial genomes previously used [55] to evaluate parsimonious gene order approaches. This is a set of 10 complete metazoan mitochondrial genomes [57] with 36 common genes. It contains two nematodes, two mollusks, two arthropods, two echinoderms, one annelid and one chordate [55]. GRAPPA was previously shown to have run for more than 48 hours without suggesting a phylogeny for this dataset [55]. MGR generated a tree in agreement with estimated phylogenetic relationships except the clustering of two arthropod genomes [55]. Our approach resulted in a clustering that tightly clustered the two arthropods in the dataset together and similarly clustered other metazoan genomes in broad agreement with the estimated phylogeny [58] with the single annelid genome as an outgroup (Additional data file 8).

The primary limitations of existing approaches are speed and data size (typically only a few hundred markers). In contrast, this study utilized over 14,000 markers (Additional data file 2) to suggest a phylogeny within a few minutes and complete ancestral gene order inference in approximately 10 minutes for cases where other methods do not converge on a solution in any reasonable amount of time. While other approaches, like GRAPPA, require gene order and orientation information along a single chromosome, this approach accommodates incomplete assemblies of multi-chromosomal genomes. The order and orientation of assembly scaffolds need not be known. Additionally, by encoding contig and scaffold edge markers and arm level indexing, one can glean valuable insights despite assembly gaps.

While this method provides a simple approach for inferring evolutionary relationships, rearrangement phylogeny, inversion count estimates, and ancestral gene order, we recognize some of its limitations. In order to overcome some of the limitations inherent in parsimonious approaches [23] (see Materials and methods) a number of practical biological assumptions are used. To ensure valid inferences at ancestral states, constraints are enforced at each ancestral state on the maximum number of pairs that a gene can be part of. Despite the fact that novel ancestral adjacencies, other than those in the input set, cannot be inferred, it has been shown that a high percentage of the total known gene count is assembled into ancestral syntenic blocks. Using the high-quality gene annotation of a single fly species (*D. melanogaster*) potentially introduces a bias in this analysis as a result of lineage-specific genes. In order to overcome this problem, the set of genes (protein coding segments in our case) that have homologs in all fly species are used, approximating equal gene content. Given that a majority of fly genes are shared across all fly species, this covers a large percentage of the known genes. As additional gene models for other fly species become available, they should be included in this analysis. This will also account for correctly quantifying gene gain and loss factors. Furthermore, homologous genome markers, other than protein coding genes, could also be used. This analysis can provide information identifying the areas of missing assembly data and positions of likely errors. In fact, under a small set of reasonable assumptions, the approach can suggest corrections to incomplete genomic assemblies. However, as is the case with any draft assembly, genome assembly errors are expected to be a factor in this analysis. Progressive cleanup of the genome assembly will lead to better results. This method potentially has some of the same limitations as other approaches associated with incorrect identification of homologous genes in the presence of paralogs. This has been addressed by selecting one member of each gene family as the best homolog (in the case of paralogs) based on local gene context and gene structure. It should be noted that the technique used in deriving rearrangement break counts could easily be translated to compute inversion counts along a branch.

While deriving phylogenetic relationships among a set of species, the rationale used by the NGP approach is based on maximizing arm-indexed 'exclusively shared NGPs' (see Materials and methods). Although such constructs can increase certainty about tree topology, inferring branch lengths from rearrangements should be treated with caution as evolutionary rates of rearrangement might differ among lineages [59]. While arm-indexing of NGPs results in a powerful tool for grouping species that share transposition events like the pericentric inversion in *D. yakuba *and *D. erecta *[47], it is prone to limitations of assembly errors or single-species transpositions involving a large number of NGPs. Assembly errors that incorrectly join scaffolds belonging to different Muller elements might result in NGPs being assigned an incorrect arm-index based on majority homolog presence on the super-scaffold. Such inaccuracies can lead to incorrect phylogenetic partitioning. Additionally, a large number of real transposition or other rearrangement events in a single species could lead to different phylogenetic groupings based on the total number of NGPs involved in such events. If that total rivals the number of NGPs shared (exclusively) with a cluster of evolutionarily close species, it would result in the placement of this species outside the cluster. An extension of this study showed that the placement of *D. willistoni *differed from the classical *Drosophila *phylogeny [32] and from studies involving mutation clocks [60]. Based on NGP analysis, after compensating for incorrect assembly joins, *D. willistoni *was placed as an outgroup species to the set of all genus *Drosophila *species under consideration (data not shown). Additional analysis with *SRP54 *and *SRP19 *protein sequences using parsimony and maximum likelihood approaches showed mixed results where one agreed with NGP phylogenetic partitioning (data not shown). Alternative NGP clustering solutions (see Materials and methods) and the relative number of gene pairs involved (an indicator of the strength of clustering) could be used in conjunction with gene tree results to select a candidate phylogeny amongst a set of close alternatives suggested by the NGP approach.

**Figure 5:**
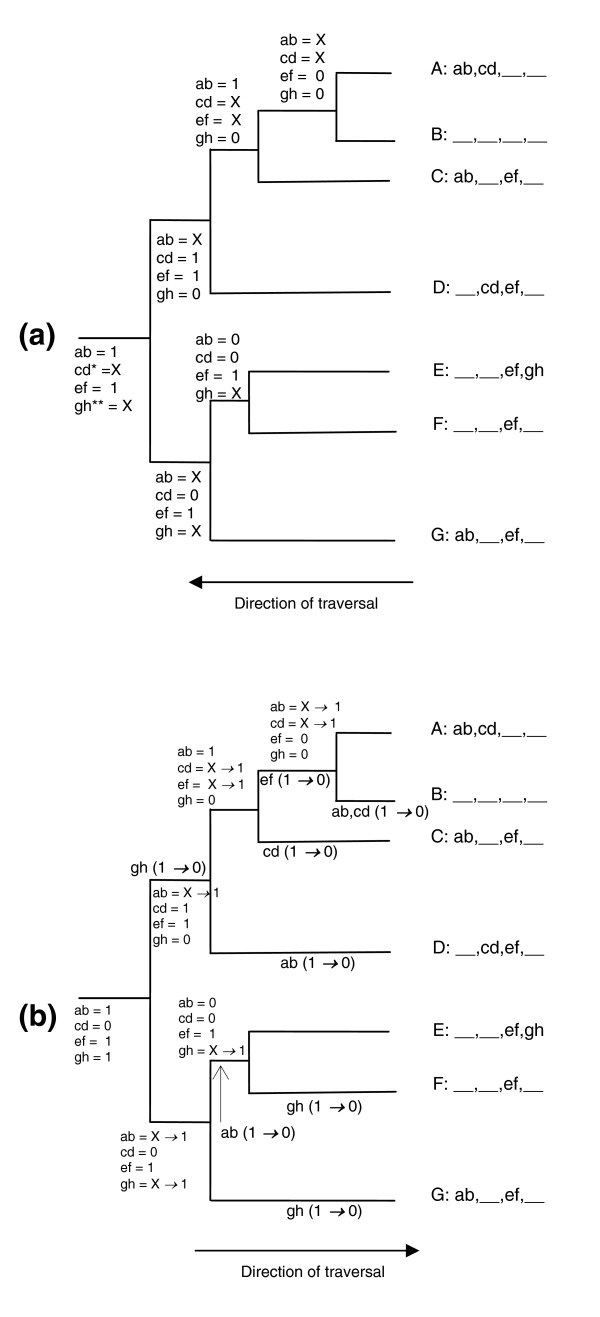
Two-stage tree traversal algorithm example. Species A through G are shown with representative gene pair content (four pairs: ab, cd, ef, gh; an underscore '_' implies that that pair does not exist in that species). The state of pairs at each node is shown and state transitions are shown in bold font. **(a) **Leaf-to-root traversal. Ancestral states of gene pairs are assigned with the constraint that a gene can be in at most two pairs at any given node. A '1' implies that the pair exists at a given node where at least one species on either side of the node has that pair. A '0' implies that it does not exist in any leaf species reachable from that node. An 'X' implies that the state is unknown due to conflicting 1/0 or X/0 information from child nodes (that is, a '1'/'X' exists for that pair on one side of the node and a '0' on the other side). 0 → X, 1 → X, and X → 1 transitions are seen during this leaf-to-root tree traversal. In the case of pairs like cd*, where a '1' and '0' are inferred at the child nodes at the root of the tree, and there is no further evidence from outgroup species, the state is left undetermined and does not contribute to rearrangement analysis. It is hoped that addition of more genomes in this analysis will help resolve this in the future. In cases where the root value is 'X' (as in pair gh**), it is set to '1' if an outgroup species has this pair (given that it already exists in at least one non-outgroup species), else it does not contribute to this analysis. **(b) **Root-to-leaf traversal. Pair gh is assumed to be set to '1' at the root of the tree for this example, using the criteria above. Rearrangements are assigned to tree branches. A 0 → 1 transition reflects creation of a pair that did not exist at an ancestral state, including pairs unique to a species. A 1 → 0 transition represents a pair being lost due to a rearrangement. X → 0 and X → 1 transitions at nodes represent inheritance of an inferred ancestral state where the current value is unknown due to conflicting child evidence. The rearrangement phylogeny counts the number of 1 → 0 transitions (NGP disruptions) along each branch. See Additional data file 10 for a detailed description of the method.

While inferring rearrangement counts, the method performs well for a set of closely related species where a large majority of the genes are conserved across all species. For example, within genus *Drosophila*, there are a large number of shared genes that result in a strong signal. However, as additional evidence is added from evolutionarily distant species, lack of a strong signal (absence of homologous genes, presence of a large number of rearrangement events leading to the outgroup species, lack of a large number of shared NGPs) limits the utility of such evidence. At the root of the *Drosophila *tree (Figure 5), for example, NGPs that have conflicting evidence from the subgenus *Sophophora *and subgenus *Drosophila *sides of the tree would normally be resolved by the algorithm with evidence from outgroup species. However, the large evolutionary distance of the outgroup species used in this study provides a diluted NGP signal, due to a large number of rearrangements along that branch. For example, only 2% of the ambiguities at the genus *Drosophila *root could be resolved with evidence from outgroup species (NGP evidence from at least one outgroup species and one *Drosophila *species). A number of ambiguities that could probably be resolved to be a '1' at the root remain unresolved. As a result, one of the limitations of this method is that it undercounts the number of rearrangement breaks at the branches close to the root of the tree (of closely related species) due to diluted signal from outgroup species (Figures 3 and 5).

## Conclusion

This approach has been shown to outperform existing techniques with its speed and ability to handle genome-scale datasets far exceeding current limitations. The ability to handle multi-chromosomal datasets with thousands of markers, the use of 'exclusive shared NGPs' for clustering, the use of arm indexing to amplify the signal between closely related species, accommodations for genome assembly incompleteness, and the two-stage tree traversal with biologically relevant assumptions to infer ancestral states are the primary features of this method. The results place major aspects of the currently believed evolutionary relationships among different *Drosophila *species on a solid footing based on full-genome comparative analysis. The clustering supports the placement of *D. yakuba *based on a large set of markers (over 14,000). This analysis has, for the first time, provided an accurate lower bound for the number of chromosomal rearrangements that might have occurred among these species since their last common ancestor. With a sequence of decreasing stringency assumptions, a set of likely ancestral syntenic gene clusters of increasing size has been inferred. With the availability of additional fly and insect genomes, this analysis can be easily extended to include additional evidence to refine the results.

## Materials and methods

One of the important assumptions exploited in this work is that chromosomal inversions in a given nucleotide sequence are rare events that result in the disruption of two pairs of neighboring genes and that the likelihood of the same inversion taking place independently along disjoint lineages is low. Neighboring pairs of homologous genes (NGPs) showing the same pair-wise orientation in distant species are considered to have escaped rearrangements via genomic inversions. Furthermore, despite the large number of theoretically possible gene pairs formed by over 8,000 genes, in practice only a fraction of this set is seen across all species. It is assumed that the probability of an inversion creating a NGP from an ancestral gene order is small, and smaller still if the NGP is seen across multiple species. In other words, a NGP found to exist in multiple species is assumed to have existed in the common ancestor, thus maximizing the similarity between extant species to derive an ancestral state.

The method outlined below falls into the general class of parsimonious gene order methods [23,61] used for phylogenetic analysis, with extensions based on our assumptions mentioned above. Most phylogenetic optimization approaches are known to be NP-hard, including the breakpoint median problem [21,23,54]. Similar to some previous approaches [61,62], we reduce the set of genes to a binary encoding based on gene adjacency. We overcome some of the known limitations of parsimonious gene order approaches with a number of simplifying biological assumptions, which prove to be practical. These assumptions, related to constraints on ancestral states, varying gene content, and ortholog identification, are outlined below. We extend previous techniques to include orientation and chromosome arm (*Drosophila *Muller element) information. We then infer a phylogenetic tree topology via clustering of species to maximize shared pairs unique to a cluster. Following this, we estimate rearrangement counts as described below. In contrast to the maximum parsimony on binary encodings (MPBE) approach [61,62], we have added arm indexing information to strengthen the signal between closely related species, and clustering is based on 'exclusively shared NGPs' between groups of species rather than straightforward parsimony analysis on encoded sequences.

All pairs of adjacent orthologous genes are identified across a set of eight fly species and four outgroup species. Beginning with *D. melanogaster*'s release 4.3 annotated gene set [44,63], genome sequences for seven other fly species [64] (version CAF1: comparative analysis freeze 1) and four outgroup species (*A. gambiae/Agam *[65,66], *Aedes aegypti/Aaeg *[67,68], *Apis mellifera/Amel *[69,70], *Tribolium castaneum/Tcas *[71]) were used. The seven *Drosophila *species used, other than *Dmel*, are: *D. yakuba *(*Dyak*), *D. erecta *(*Dere*), *D. ananassae *(*Dana*), *D. pseudoobscura *(*Dpse*), *D. virilis *(*Dvir*), *D. mojavensis *(*Dmoj*), *and D. grimshawi *(*Dgri*). This potentially large data set of adjacent gene pairs was stored in a simple and compact binary data structure. A simple parsimonious clustering based on maximizing the number of common gene pairs unique to a cluster was performed in order to identify a phylogenetic tree. Unique gene pairs point to rearrangements specific to a species. A two-stage iterative procedure that walks from the leaves of the *Drosophila *tree to the root and back to the leaves was then used to infer rearrangements along specific branches of the phylogenetic tree. It was also used to infer syntenic blocks (gene ordered clusters) at various nodes in the tree, including the root of the genus *Drosophila *tree, using a set of progressively relaxed criteria. The resulting dataset gives a probable ancestral gene arrangement and syntenic block structure at the root of the *Drosophila *tree. The key steps of this method are outlined below and detailed in Additional data file 10.

### Homologous gene identification

In each species, genes homologous to the reference set (*D. melanogaster*) are identified while accommodating for assembly gaps [72]. This was done using standard sequence comparison methods to maximize sequence similarity, including tBLASTn, along with techniques to distinguish orthologs from paralogs due to gene duplication. Neighboring gene context was also used to identify homologs, which we recognize adds some circularity as NGPs are later used to create syntenic blocks. It should be noted that, although homologous genes have been used in this analysis, the method presented here is applicable to a wide range of homologous genome markers for which homology between species can be determined. This includes non-protein coding genes, micro-RNAs and transposable elements.

### Adjacent gene-pair classification

Using homologous gene sets for each species, pairs of adjacent genes are recorded based on their mutual orientation (direction of transcription). A pair can include two adjacent genes, in a specific order, that are: convergent (→←), divergent (←→), or transcribed in the same direction (→→ and ←←). The mutual order of transcription starts and ends is important in determining equivalence between pairs. In order to accommodate for gaps in genome assembly, genes found at the edges of assembly scaffolds are recorded as part of special pairs (_←, _→, ← _, → _). Finally, scaffolds with a single gene hit are also noted (_← _, _→ _).

### Data structure

The data structure used to capture gene adjacency information is a five-dimensional binary matrix representing the presence or absence of a given gene pair in a given species with location and directionality also encoded:

M*_i, *j*, *o*, *s*, *m *_= {0,1}

where *i*, *j *= genes i and j; *o *= one of the gene pair orientations identified above; *s *= a given species; *m *= the arm index for the gene pair. A '1' implies that a gene pair consisting of adjacent genes *i *and *j *in a specific pair-wise orientation *o *exists in species *s *on the chromosomal arm (*Drosophila *Muller element) encoded by *m*. A '0' implies that it does not. Assembly scaffold edges and single gene scaffolds can be included as special case gene markers. Given the symmetric nature and sparse data content of this matrix, standard storage optimization techniques can be used to reduce the size of the stored binary data. Chromosomal arm encoding is typically applicable and useful in resolving relationships between close species that share the same chromosome architecture. For comparisons with outgroup species with different chromosomal architecture, this indexing requirement can be relaxed as NGP differences will dominate due to evolutionary divergence. Using this basic data structure, binary encoded arrays to aid easy lookup of NGPs across species can be devised (Additional data file 10).

### Phylogenetic reconstruction via clustering

Using a simple hierarchical clustering approach, pairs or groups of species are clustered in order to maximize the number of shared gene pairs unique to the clustered group ('exclusively shared NGPs'). This is based on the idea that species that share an evolutionary lineage possess (or lack) a number of identical inversions and hence share NGPs unique to the group. With the option of arm-indexed NGPs, this approach also allows for the clustering of groups of closely related species in smaller clusters although they might share NGPs in common with other species farther away. Alternatively, species could be clustered based on total number of common shared pairs (not necessarily unique to the grouping). Clustering continues until a binary partitioning of the species, based on decreasing cardinality, is obtained. A simple validation of this clustering was performed using a gene tree (Figure 2) generated with PHYLIP [73] using coding sequence predictions for *SRP54 *and *SRP19 *genes for *Drosophila *species [64] (Drosophila 12 Genomes Consortium, 2007).

Any intermediate results that violate previously clustered boundaries can be analyzed for alternative or weak relationships between species. For example, in the arm-indexed clustering results for these species (Additional data file 4), the first violation with 463 NGPs shows that *D. melanogaster *shares a significant number of NGPs with all the flies except *D. yakuba *and *D. erecta*. These three species were previously clustered together (544 NGPs). *D. yakuba *and *D. erecta *should account for the disruption or translocation of 463 NGPs. This is borne out by the strong clustering of *D. yakuba *and *D. erecta *(751 NGPs) where the underlying translocation and inversion events account for the disruption of NGPs previously shared with *D. melanogaster*. The second violation with 357 NGPs (found in all other species except *D. pseudoobscura*) points to the fact that *D. pseudoobscura *exhibits a large number of taxon-specific inversion events (and hence unique NGPs). This is also borne out by the first line labeled as a 'leaf', which counts the actual number of *D. pseudoobscura *specific unique NGPs (937) derived from this dataset. Analysis of non-arm-indexed NGP clustering results (Additional data file 5) shows alternative clusters for the *D. melanogaster*, *D. yakuba and D. erecta *trio: *D. melanogaster + D. erecta *(16 NGPs), *D. yakuba + D. erecta *(15 NGPs), and *D. melanogaster + D. yakuba *(9 NGPs). Arm-indexed clustering shows a strong signal reflecting an underlying shared pericentric inversion and selects the second of the three solutions above.

### Rearrangement counts along various evolutionary paths

The rearrangement phylogeny estimates the number of inferred ancestral NGP disruptions along a branch of the evolutionary tree. An estimate of the inversion count can be computed from a rearrangement phylogeny as the number of inversion events that lead to the observed rearrangements (each inversion disrupts two ancestral gene pairs and creates two new pairs). Once the phylogenetic relationships have been derived, a two-stage tree traversal methodology can be used to infer the rearrangement phylogeny. The arm level indexing criteria is relaxed at this stage in order to allow NGPs on different arms to contribute to ancestral gene order inference. This allows the consideration of pericentric inversions or segmental transpositions in this method. This process is summarized with a simple example (Figure 5). First, a tree traversal from the leaves to the root can be used to infer the NGPs that are in common between each ancestral node and its child nodes, based on our heuristic of maximizing the similarity between extant species at any ancestral node. An ancestral node where any two leaves reachable from that node along disjoint paths show the same NGP is assumed to have had that NGP in its sequence. The motivation behind this heuristic is the assumption that an NGP that exists in at least one species on either side of a node exists at that node, and the likelihood of independent inversions creating these pairs in different species is low. Conflicting evidence from two child nodes suggesting that rearrangements might have taken place along the path to a child are noted. These ambiguities are resolved locally with the next species along the hierarchy or higher up in the hierarchy, including using outgroup species information. In cases where a node is inferred to have one NGP corresponding to one pair of a two break inversion event relative to a neighboring species, the other pair can be inferred to exist at the node if its assignment is ambiguous. Additionally, a gene is constrained to be part of, at most, two pairs at any given node. Ambiguities in determining the NGPs at the root of the genus *Drosophila *tree can be resolved by using an outgroup species as far as possible. After the leaves-to-root traversal is done, a traversal from the root to leaves is initiated. During this process, any remaining ambiguities existing at internal nodes are resolved by inheriting the ancestral state of an NGP wherever possible. Rearrangement counts along each branch can be estimated by counting the number of cases where an NGP exists at a given node, but does not exist at a child node. This gives the rearrangement phylogeny and an alternative estimate of the branch lengths of the phylogenetic tree.

Rearrangement events are the result of inversions that disrupt two NGPs present at the ancestral state (and create two new NGPs). Inversions along various paths can be counted using the fact that four pairs (two disruptions and two creations) are involved in an inversion. Thus, the disrupted pairs counted in the rearrangement phylogeny can be divided by two to get an estimate of the inversion count. It is possible to extend this analysis to include a correction factor to account for the impact of rearrangement breakpoints being reused based on varying reuse rates between species.

In cases where only a single species on either side of a node has an NGP that is absent from all other species, a rearrangement break for this NGP would be assigned to all top level internal branches that lead to other species clusters (and to leaf branches in the same cluster as the species having the NGP). The extreme case would be where there are a large number of species on both sides of the node. Although this is expected to be a rare occurrence, genes on the edges of genomic hotspots can contribute to this phenomenon.

### Ancestral syntenic block inference

Chaining together NGPs that share a gene in common (in the right orientation), or bridging blocks using existing NGPs, ultimately leads to a set of ancestral syntenic blocks at the root of the *Drosophila *tree. We use a set of progressively relaxed stringency assumptions. First, we recursively chain together NGPs that have a gene in common (in the right orientation). A gene is restricted to be part of two pairs at most, so conflicts have been resolved at the earlier stage to determine the existence of an NGP at a node. This process will generate an initial set of syntenic blocks of genes at an ancestral node. Extending blocks with NGPs or other blocks into larger entities can only be done if the NGP used to bridge them matches the mutual orientation of the genes on the edges of these blocks. It is occasionally necessary to flip a block or NGP for this to be feasible.

The criteria for forming and enlarging syntenic blocks can be progressively relaxed based on the assumption that the probability of independent inversion events bringing together a particular pair of genes in disjoint lineages is rare. The various criteria are as follows.

#### Criterion 1

This is the case where the procedure described in the example (Figure 5) is used to determine NGPs that exist at the *Drosophila *root and these NGPs are used to form syntenic blocks as described above.

#### Criterion 2

For criterion 2, extend syntenic blocks by recursively chaining together blocks whose edges appear in a gene pair in at least one fly species and an outgroup species with the correct mutual orientation. Flipping of NGPs or blocks might be necessary to accomplish this.

#### Criterion 3

For criterion 3, extend syntenic blocks by chaining together blocks whose edges appear in a gene pair in at least one fly species in the desired orientation. In the case of assembly gaps, this also covers cases where a gene might be on the edge of a scaffold in multiple species. This strategy sometimes leads to joins of lower confidence, as in some cases a block might be a candidate for merging with two separate blocks with conflicting evidence from individual species. In the absence of additional information, such joins made with an arbitrary choice between alternative blocks can be tagged as low-confidence joins.

### Identifying genomic regions of increased rearrangement activity

This approach leads to the straightforward identification of pairs of genes where each individual gene is found in multiple dissimilar pairs across all species. By using a reasonable threshold of a number of pairs, where each gene is part of that many dissimilar pairs, a set of genes bordering probable regions of high rearrangement activity can be obtained. It should be noted that the identification of such regions does not directly imply rearrangement hotspots with resolution at the nucleotide level.

## Abbreviations

*Dana*, *D. ananassae*; *Dere*, *D. erecta*; *Dgri*, *D. grimshawi*; *Dmel*, *D. melanogaster*; *Dmoj*, *D. mojavensis*; *Dpse*, *D. pseudoobscura*; *Dvir*, *D. virilis*; *Dyak*, *D. yakuba*; NGP, neighboring gene pair; TSP, traveling salesman problem.

## Authors' contributions

AB and TFS contributed to the design and implementation of the algorithm, the analysis and interpretation of the data, and to drafting and revising the manuscript. WMG contributed to the analysis and interpretation of the data and to revising the manuscript.

## Additional data files

The following additional data are available with the online version of this paper. Additional data file 1 is a filtered list of genes common to all *Drosophila *species in set (high confidence placements). Additional data file 2 lists NGPs based on common gene set (in file 1) with arm indexing and only within *Drosophila *species (without outgroup pairs) - used in clustering of fly species (Figure 1a). Additional data file 3 list NGPs without arm indexing (to allow for NGPs from outgroup species with varying chromosome architecture) based on *Drosophila *common gene set (in file 1) - used in the rearrangement and ancestral gene order analysis. Additional data file 4 provides clustering results for Figure 1(a). Additional data file 5 provides clustering results for Figure 1(b). Additional data file 6 shows ancestral adjacencies (blocks) at the *Drosophila *root under criterion 1. Additional data file 7 shows ancestral adjacencies (blocks) at the *Drosophila *root under criterion 2. Additional data file 8 is a summary of results of testing with the mitochondrial test dataset. Additional data file 9 is a Tree file from PHYLIP version 3.65 for *SRP54 *and *SRP19 *gene sequences used for Figure 2. Additional data file 10 gives a detailed description of the method. The code is available from the authors, upon request.

## Supplementary Material

Additional data file 1 Filtered list of genes common to all *Drosophila *species in set (high confidence placements). Click here for file

Additional data file 2 NGPs based on common gene set (in file 1) with arm indexing and only within *Drosophila *species (without outgroup pairs) - used in clustering of fly species (Figure 1a). Click here for file

Additional data file 3 NGPs without arm indexing (to allow for NGPs from outgroup species with varying chromosome architecture) based on *Drosophila *common gene set (in file 1) - used in the rearrangement and ancestral gene order analysis. Click here for file

Additional data file 4 Clustering results for Figure 1(a) Click here for file

Additional data file 5 Clustering results for Figure 1(b). Click here for file

Additional data file 6 Ancestral adjacencies (blocks) at the *Drosophila *root under criterion 1 Click here for file

Additional data file 7 Ancestral adjacencies (blocks) at the *Drosophila *root under criterion 2. Click here for file

Additional data file 8 Summary of results of testing with the mitochondrial test dataset. Click here for file

Additional data file 9 Tree file from PHYLIP version 3.65 for *SRP54 *and *SRP19 *gene sequences used for Figure 2. Click here for file

Additional data file 10 Detailed description of the method. Click here for file
