# Supplementary material for: Inferring genome-scale rearrangement phylogeny and ancestral gene order: a Drosophila case study
Source: Genome Biol. 2007 Nov 8;8(11):R236. doi: 10.1186/gb-2007-8-11-r236 (PMC2258185; doi:10.1186/gb-2007-8-11-r236)

Supplementary material with results using mitochondrial DNA test set

**[Gene order bu Jeffery L. Boore at:
http://evogen.jgi.doe.gov/cgi-bin/Mt_gene_order_browser.cgi]**

Species and Metazoan group codes used:
Onchocerca volvulus (Nematodes) : O1(NEM)
Ascaris suum (Nematodes) : A1(NEM)
Lumbricus terrestris (Annelids) : L1(ANN)
Cepaea nemoralis (Mollusks): C1(MOL)
Albinaria caerulea (Mollusks): A2(MOL)
Artemia franciscana (Arthropods): A3(ART)
Drosophila yakuba (Arthropods) : D1(ART)
Strongylocentrotus purpuratus (Echinoderms) : S1(ECH)
Asterina pectinifera (Echinoderms): A4(ECH)
Homo sapiens (Chordates): H1(CHO)

Notes

- mtDNA with equalized gene content results in 36 genes
- See supplementary files containing original mtDNA gene order and one encoded with integers.
- There are two competing clustering solutions that differ in the placement of Chordates (H1 CHO). Both are very close – differing only in a single common pair (NGP).
- Both solutions correctly cluster the Nematodes, Mollusks, Arthropods, and Echinoderms with the Annelid left over as the outgroup.
- Solution #2 (alternate 2 below) matches the currently understood metazoan phylogeny [Boore J and Brown W in Mol. Biol. Evol. 7: 87-106, 2000]
- Katharina tunicata (MOL) is dropped from the analysis set as it is known to show weak association with other mollusks as outlined in [Blanchette M, Kunisawa T, Sankoff D in J. Mol. Evol. 49: 193-203, 1999]

The following pages contain:

- Clustering solutions for these species based on NGP analysis
- Derived tree phylogeny based on above binary clustering

- **Binary clustering alternate 1**
  Numbers represent the shared NGPs exclusive to a grouping.

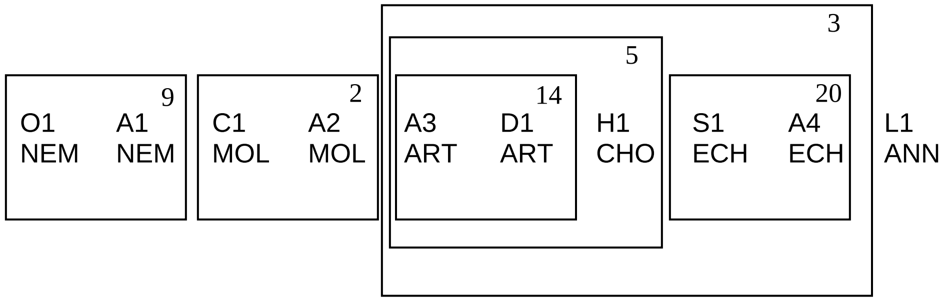

- **Binary clustering alternate 2**
  Numbers represent the shared NGPs exclusive to a grouping.

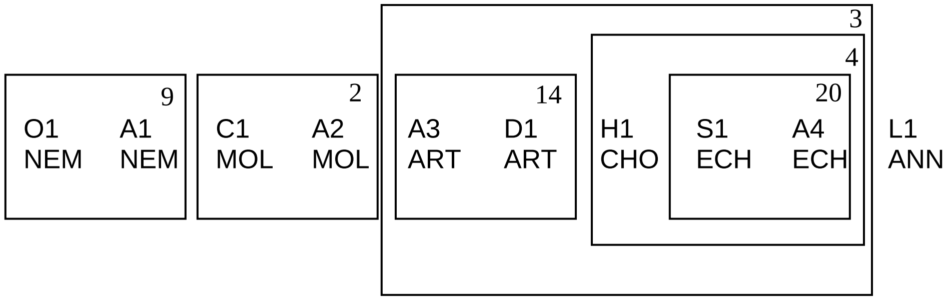

- **Derived Phylogeny for alternate 1**

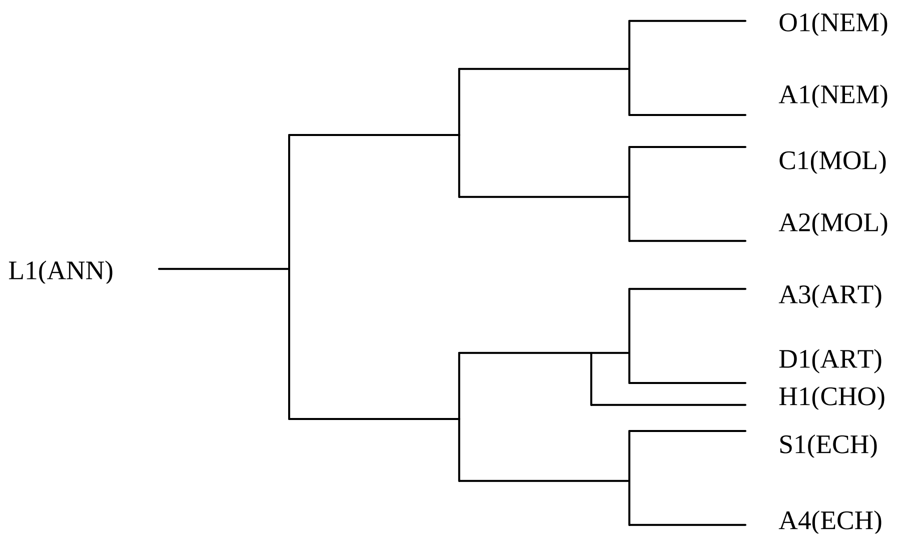

- **Derived Phylogeny for alternate 2**

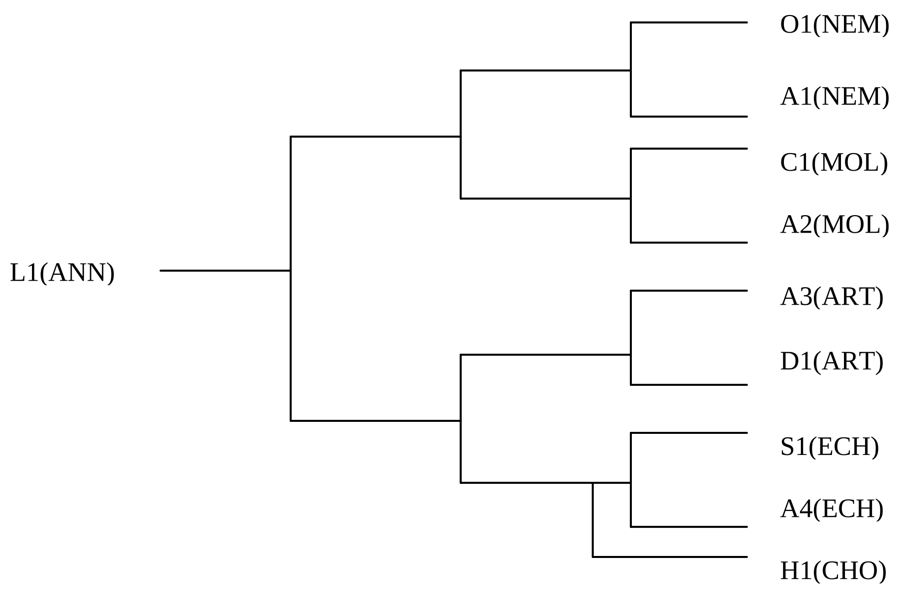

Supplement: Additional data file 8 — Summary of results of testing with the mitochondrial test dataset. [file gb-2007-8-11-r236-S8.pdf]
