# Supplementary material for: Inferring genome-scale rearrangement phylogeny and ancestral gene order: a Drosophila case study
Source: Genome Biol. 2007 Nov 8;8(11):R236. doi: 10.1186/gb-2007-8-11-r236 (PMC2258185; doi:10.1186/gb-2007-8-11-r236)
Supplement: Additional data file 10 — Detailed description of the method. [file gb-2007-8-11-r236-S10.pdf]

Supplementary File 9: Description of NGP processing

1. **Input:**

    a. Set of genetic markers from a reference species (***S_1***) annotation (in this case *D.melanogaster* protein coding gene set).
    b. Set of candidate species (***S_2…S_L***) assemblies with homologous locations for each marker from the reference set. Paralog and duplication issues (for proteins) cleaned up via synteny processing (similar to [1]) to obtain the best syntenic location for a homolog.
    c. If assembly is not fully assembled into units at the chromosomal level (chromosomes/arms ***A_1…A_m***), the scaffold sizes and scaffold-to-chromosome mapping file where available. If scaffold-to-chromosome mapping data is not available (preliminary assembly), it can be inferred from majority hits of proteins from the reference species (this is useful for species like Drosophila where single gene arm translocations are rare events).
    d. A set of one or more outgroup species with homologous locations marked – used to resolve ambiguities at the root of tree for species (***S_1…S_L***). Outgroup set is not used in arm-indexed clustering as outgroup chromosomal architecture might differ.

2. **Data Pre-processing (description assumes protein coding gene set as markers)**

    a. Determine set of proteins ***P_1….P_n*** where for each protein ***P_k*** $\in$ { ***P_1….P_n***}:
        i. ***P_k*** has a homolog assignment in at least ***s*** of the ***L-1*** candidate species. In this implementation ***s=L-1.*** **Note:** Outgroup species are not part of this set.
        ii. ***P_k*** is not an embedded gene.
        iii. ***P_k*** is not part of a tandem repeat cluster of homologous genes where the exact order of homologs cannot be discerned.
    b. Record the chromosome arm (one of ***A_1…A_m***) for each homologous location in each species.
    c. For each chromosome arm (or scaffold) in each species:
        i. Identify pairs where makers are adjacent to each other. For example, the order ***a-b-c*** results in the pairs ***ab*** and ***bc*** in a given species. The total number of unique marker pairs across all species is ***N***.
        ii. Identify the mutual orientation (example: direction of transcription for genes) of markers in each of these pairs as:
            1. ***O_1***: convergent (→ ←)
            2. ***O_2***: divergent: (← →)
            3. ***O_3***: same direction: (→→, ← ←)

iii. Record markers on the edges of scaffolds as part of a single marker-pair with the preceding marker. Single markers on a scaffold are not recorded as part of a marker-pair.

3. **Populate NGP Data Structure**
   a. Five dimensional binary matrix (***M****) shown below where a bit value of "1" implies that the marker pair (in that mutual orientation, with that arm index, in that species) exists.

$$M^*_{i,j,o,s,m} = \{0,1\}$$

where i , j = Markers i,j $\in \{P_1 \ldots P_n\}$
o = Mutual orientation o $\in \{O_1 \ldots O_3\}$
s = Species s $\in \{S_1 \ldots S_L\}$
m = The Muller arm index for gene pair, m $\in \{A_1 \ldots A_m\}$

   b. For ease of lookup, populate a binary string array (***M***) indexed by NGPs that represents the presence/absence of that NGP in a list of species (ordered list). Length of each entry in ***M*** equals the number of species: ***L***. The number of entries in ***M*** is ***N*** (defined above). (Note: With slight modifications to a few steps, ***M*** can be populated directly and ***M**** can be skipped.)

$$M_{i,j,o,m} = \{0,1\}_L$$

where i , j = Markers i,j $\in \{P_1 \ldots P_n\}$
o = Mutual orientation o $\in \{O_1 \ldots O_3\}$
m = The Muller arm index for gene pair, m $\in \{A_1 \ldots A_m\}$

4. **Phylogenetic relationship via Species Clustering**

   a. Group by binary string value in $M_{i,j,o,m}$
   b. Each group includes the NGPs where the value of the binary string is identical i.e. where $M_{i,j,o,m} = M_{i',j',o',m'}$
   c. Sort the results of this grouping of binary strings $b_1 \ldots b_L \in M$ in decreasing order based on the strength of the grouping (number of NGPs that have this binary string value in ***M***). Each group represents the number of "exclusively shared NGPs" (arm-indexed) within those species where $b_x = 1$, $x \in (1..L)$, in the binary string. These are NGPs (with identical characteristics) found in all of these species and not found in any other species (where $b_x = 0$) .
   d. Evaluate each group in decreasing strength of the grouping (defined above; MS-XL):
      i. Hierarchically cluster species that have $b_x = 1$ in the binary string representing this group.
      ii. Allow sub-clusters within clusters (and larger clusters of sub-clusters). Mark any clusters that cross boundaries of previously defined clusters for later evaluation as alternate solutions.

iii. Stop clustering of species once a binary partitioning is obtained. This corresponds to the inferred phylogenetic relationship between these species.

5. **Leaf-to-Root Walk (Inferring ancestral adjacencies and conflicts)**

   a. The arm-index (m) in $\boldsymbol{M}_{i,j,o,m}$ is relaxed from here on for this method such that NGP disruptions are identified regardless of translocations, arm fusions etc. So, $\boldsymbol{M}_{i,j,o,m} = \boldsymbol{M}_{i,j,o,m'}$ from here on.
   b. This also allows for the inclusion of NGP evidence from outgroup species where chromosomal architectures might be different.
   c. For the binary tree corresponding to the hierarchical clustering of species, consider each non-leaf node at each level (going from nodes closest to the leaf nodes towards the root of the tree – bottoms-up tree traversal). All non-leaf nodes at a given level are considered before moving on to the next higher level.
   d. For each ancestral node and its two child nodes:
      i. For each of $\boldsymbol{N}$ NGPs, derive a value $\in$ {1,0,X} for that ancestral node:
         1. Assign "1" if child nodes are (1,1) OR (1,X) OR (X,X); Do not assign "1" if the marker is already part of 2 NGPs which have a value of "1" at that node.
         2. Assign "0" if child nodes are (0,0)
         3. Assign "X" if child nodes are (1,0) OR (X,0)
         4. At the root of the tree, assign a "1" to any "X" that has a "1" value for that NGP in an outgroup species (X implies that at least one species in the non-outgroup tree has that pair)

6. **Root-to-Leaf Walk (Resolving conflicts and inferring fixed rearrangement break counts)**

   a. Starting at the root of the species ($\boldsymbol{S_1 \ldots S_L}$) tree and moving towards the leaf nodes (top-down tree traversal) – consider each ancestral node before its child node is considered.
   b. For each NGP at each ancestral node:
      i. For a given NGP, if any of the child nodes has a value of "X", assign it the value of the ancestral node if $\in$ {0,1}. Do not perform X→1 update if the marker is already part of 2 NGPs which have a value of "1" at that node.
      ii. After updating values of all such NGPs, count the number of 1→ 0 value transitions from an ancestral node to each of its child nodes. Record that value as the number of NGP ($\boldsymbol{D_{ab}}$) disruptions along that path. $\boldsymbol{D_{ab}}$ = number of ancestral NGP disruptions going from node a to child b.

c. Leaf nodes do not have an "X" value, but only 1,0 values in the binary matrix. For these, count the number of 1→ 0 transitions from the ancestral node.
d. Record 1→ 0 transitions for NGPs from the root of the ($S_1 \ldots S_L$) species tree to the outgroup species: D_{ro}

7. Ancestral Synteny Inference

a. At the root of the species ($S_1 \ldots S_L$) tree use the following relaxed criteria:
   i. *Criterion 1*: Recursively join pairs (or chains of marker pairs) into longer chains where markers on the edges of two pairs/chains are the same and exist in the same orientation such that mutual orientation of NGPs is not affected. Only one copy of the marker is retained in the longer chain.
   ii. *Criterion 2***:** Supplement Criterion 1 to join pairs/chains where the markers (***i,j***) on the edges form a "bridging pair" in a particular orientation that has $M^*_{i, j, o, s, m} = \{1\}$ for at least one species $s \in \{S_1 \ldots S_L\}$ AND for one outgroup species.
   iii. *Criterion 3***:** Supplement Criterion 2 to join pairs/chains where the markers (***i,j***) on the edges of existing pairs/chains form a "bridging pair" in a particular orientation that has $M^*_{i, j, o, s, m} = \{1\}$ for at least one species **s** $\in \{S_1 \ldots S_L\}$.

8. Output

a. Inference of phylogenetic relationships between species ($S_1 \ldots S_L$) based on "exclusively shared NGPs".
b. D_{ab} **:** The count of NGP disruptions from each ancestral node to each of its child nodes.
c. D_{ro}**:** The count of NGP disruptions from the root of the species ($S_1 \ldots S_L$) tree to the outgroup species (or root of the outgroup tree).
d. Ancestral synteny inference (chains of NGPs) for Criteria 1, 2, 3 identified above**.**

<u>**References:**</u>

1 Bhutkar A, Russo S, Smith TF, Gelbart WM: **Techniques for Multi-Genome Synteny Analysis to Overcome Assembly Limitations**. *Genome Informatics* 2006, **17**(2):152-161.
